# Wild jackdaws are wary of objects that violate expectations of animacy

**DOI:** 10.1098/rsos.181070

**Published:** 2018-10-24

**Authors:** Alison L. Greggor, Guillam E. McIvor, Nicola S. Clayton, Alex Thornton

**Affiliations:** 1 Department of Psychology, University of Cambridge, Cambridge CB2 3EB, UK; 2 Institute for Conservation Research, San Diego Zoo Global, Escondido, CA 92027, USA; 3 Centre for Ecology and Conservation, University of Exeter, Penryn TR10 9FE, UK

**Keywords:** animacy categories, corvid, field experiment, inanimate object, threat assessment

## Abstract

Nature is composed of self-propelled, animate agents and inanimate objects. Laboratory studies have shown that human infants and a few species discriminate between animate and inanimate objects. This ability is assumed to have evolved to support social cognition and filial imprinting, but its ecological role for wild animals has never been examined. An alternative, functional explanation is that discriminating stimuli based on their potential for animacy helps animals distinguish between harmless and threatening stimuli. Using remote-controlled experimental stimulus presentations, we tested if wild jackdaws (*Corvus monedula*) respond fearfully to stimuli that violate expectations for movement. Breeding pairs (*N* = 27) were presented at their nests with moving and non-moving models of ecologically relevant stimuli (birds, snakes and sticks) that differed in threat level and propensity for independent motion. Jackdaws were startled by movement regardless of stimulus type and produced more alarm calls when faced with animate objects. However, they delayed longest in entering their nest-box after encountering a stimulus that should not move independently, suggesting they recognized the movement as unexpected. How jackdaws develop expectations about object movement is not clear, but our results suggest that discriminating between animate and inanimate stimuli may trigger information gathering about potential threats.

## 1. Introduction

Objects in the natural world can be classified based on their potential for self-propelled motion; a fundamental divide noted by Aristotle over 2000 years ago [1]. In nature, animate objects—objects which can move of their own accord—can take a variety of forms, such as predators, social agents or moving prey. By contrast, inanimate objects make up the abiotic and biotic features of habitat and non-moving food sources. The ability to anticipate an object's potential animacy could help animals categorize environmental stimuli (i.e. differentiate them into classes), but for what adaptive purpose? In humans, such abilities have been suggested to facilitate the representation of others as intentional agents and to lead to the formation of supernatural beliefs when we misattribute cause to unpredictable events [2]. By contrast, the function of animate versus inanimate categories in guiding behaviour in other species has received less attention.

Research on responses to object animacy has mainly focused on the psychological underpinnings of animacy categories rather than their functional consequences. There is evidence to suggest that human infants and captive primates both discriminate between stimuli based on whether or not they violate expectations (i.e. spontaneous predictions of what is likely to happen) about animacy [3–5]. Similarly, other species, including species of birds, dogs and fish, selectively attend to self-propelled motion [6–8] and may show preferences for vertebrate-style movement from birth [9]. Responses to animacy and motion in both human infants and young non-humans are commonly taken as evidence for genetically evolved, implicit rules that help categorize the world [10] (sometimes referred to as ‘core knowledge’ [11]). However, it is quite possible that animals learn to categorize objects in the environment as animate or inanimate on the basis of experience that is guided by selective attention to motion. Regardless of how these categories are formed, the existence of such ingrained attentional mechanisms across vertebrates suggests that animacy categorizations may provide important adaptive benefits. This adaptive function has traditionally been framed in the context of social behaviour. For example, evidence that captive primates make animacy distinctions is often argued to reflect common, fundamental building blocks of social cognition across human and non-human primates [5]. Meanwhile, in young precocial chicks, preferences for biological motion have been suggested to aid in imprinting on their mothers [2,9]. There has been little research examining the function of animacy distinctions outside of imprinting or in non-social contexts.

To date, research on animacy categorizations in animals has been restricted to laboratory conditions, commonly using highly artificial stimuli such as point-light displays on computer screens or projections of geometric shapes (e.g. [8,9]). Although the similarity of stimuli features to naturally animate stimuli has been suggested to influence animacy attribution in captive monkeys [4], the extent to which animals categorize naturally occurring objects on the basis of animacy remains unclear. Critically, while some authors have speculated about the ecological function of animacy categorizations for animals in their natural environments (e.g. as a foundation for empathy [12]), there have been no experimental studies in the wild in which animacy norms are explicitly violated.

Understanding the ecological function of animacy categorization requires examining how such categorizations may guide decision-making in ecologically relevant contexts in the wild. One potential function that has remained experimentally unexplored is the use of animacy categories in threat assessment. The ability to rapidly attend to moving stimuli helps animals identify predators from a static background, which is why the movement is a common predatory cue [7,13]. However, to foresee potential threats and make appropriate decisions without having to wait for the movement to occur requires that animals form prior expectations about animacy—the *potential* for movement.

Birds from the corvid family (Corvidae) provide an ideal study system for testing whether or not wild animals can form expectations about animate stimuli in threat assessment contexts. Captive corvids have demonstrated knowledge of other object contingencies, such as rules governing object support [14,15] and have been argued to display similar mechanisms of physical cognition to primates [16–19]. Additionally, wild corvids have been shown to categorize different types of objects and are typically very wary of novelty [20], which may function as a way to assess unknown threats by rapidly identifying unexpected situations [21,22], potentially including those that violate expectations of movement.

**Figure 1.**
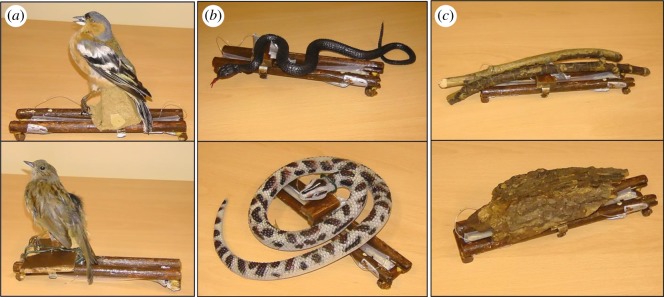
Experimental stimuli on movement tracks. The birds (*a*) were freeze-dried specimens found freshly dead in the local area. The birds, a chaffinch (*Fringilla coelebs*) and a dunnock (*Prunella modularis*), are similarly sized, non-threatening species that jackdaws commonly encounter. The snakes (*b*) were rubber toys that mirrored the patterning of two forms of a local snake species: the melanistic and the male wild-type adder (*Vipera berus*). The sticks (*c*) were found around the study sites. All stimuli could be hooked up to a hidden motor and moved along the tracks with a remote control when the fishing wire (pictured) was attached to the motor. The tracks for each stimulus are the same size (20 cm).

To determine if corvids respond to violations of animacy norms, we presented breeding pairs of wild jackdaws (*Corvus monedula*) with a series of moving and non-moving models of ecologically relevant stimuli that differed in their propensity for independent motion and in their potential threat levels. We used two different exemplars for each type of model we presented: (i) small passerine birds (non-threatening and able to move), (ii) snakes (threatening and able to move) and (iii) sticks (non-threatening when stationary, but violate animacy norms if they move independently, which should prompt further information gathering about threat; figure 1). At every object presentation when birds return to their nest-boxes, a hidden, remote-controlled motor was triggered that either moved the model or not, depending on the condition. Animacy violations were not determined by looking time preferences, as is common in laboratory studies [23], because jackdaws naturally have a wide visual field, which necessitates captive training on a peep-hole set-up. Instead, we quantified (A) whether or not wild individuals were startled at the sight of movement, (B) how their subsequent decisions (turning their back on the stimulus to enter their nest-box versus continuing to assess the stimulus) were influenced by the stimulus type and movement combination, and (C) whether or not individuals produced alarm calls at their nest during that time. Since movement is a potent cue, even when stimuli are not predatory [13], we expected jackdaws to be startled when any object moved suddenly. Additionally, we predicted that adult jackdaws would distinguish between stimuli that do and do not violate animacy expectations. If animacy violations prompt greater information gathering about risk, then distinctions between expected and unexpected movement should be reflected in longer delays before entering the nest-box. Finally, we predicted that known threats should be met with greater alarm responses, while unknown threats may deserve greater caution.

## 2. Material and methods

### 2.1. Study sites

Two jackdaw nest-box populations within the UK were tested in separate years: one in Madingley, Cambridgeshire (2014), the other across villages in Cornwall (2015). Nest-boxes were erected 4–5 m off the ground and were accessible via extendable ladders. All boxes were of the same dimensions, with a platform attached to the side of the box (see details in [24]). Daily checks verified hatching and fledging dates of each nest. Jackdaws in each population were colour-ringed in the nest or trapped as adults using ladder traps and nest-box trap doors. Colour-ringed birds could be identified individually, but not all birds in the population were colour-ringed (40% of box holders had at least one ringed bird). Additional features, such as missing feathers or tail condition, could be used to distinguish individuals within non-ringed pairs if needed. A total of 27 nest-boxes were tested in the study.

### 2.2. Experiments

Tests were conducted during the breeding season (May–June) in 2014 and 2015. The testing apparatus consisted of a camouflage-pattern cover that housed a remote-controlled motor and a set of tracks upon which the stimulus could move back and forth along a horizontal plane. To habituate jackdaws to the apparatus, an identical camouflage cover and tracks was placed on the platform of all 27 nest-boxes used in the study, at least 5 days prior to the beginning of testing. Triggering the remote control caused an internal motor to spin backwards and forwards. In *Moving* trials, where the stimulus was hooked up to the motor, triggering pulled the stimulus back and forth along the tracks at approximately 2 cm s^−1^ (electronic supplementary material, video S1, for example). The motor was triggered in all conditions, *Moving* and *Non-moving*, and made an identical noise regardless of whether or not the stimulus was attached to it.

Each box received a *Moving* and a *Non-moving* condition of each of three stimuli: a small passerine bird, a snake model and a stick (figure 1). The bird served as an ecologically plausible, yet non-threatening stimulus regardless of movement, the snake a plausible, yet threatening stimulus regardless of movement, and the stick a plausible, non-threatening stimulus when stationary, but an implausible, stimulus when moving. Two models of each type were used to prevent pseudo-replication. The birds were freeze-dried specimens of native, commonly encountered species (*Fringilla coelebs* and *Prunella modularis*). The snakes were rubber toys with similar patterns to two forms of a local venomous snake species that can climb trees: the melanistic and the wild-type adder (*Vipera berus*). Rubber snakes have been used as effective predator models in other studies of birds, including corvids (e.g. [25,26]). While *V. berus* primarily forages on small mammals, these snakes will also predate nestlings [27] and thus could represent a threat to the nest. A *V. berus* snakeskin was found at the base of a tree with one of the study's nest-boxes, confirming their presence in the area. The sticks were also both collected from the local area. The *Moving* and *Non-moving* trials of each stimulus type occurred in consecutive pairs. The presentation order within and between the pairs was pseudo-randomized between nest-boxes. Each nest-box received the same version of the stimulus type for their *Moving* and *Non-moving* trials. All six trials occurred on consecutive days where possible.

Trials occurred during the period between nestling hatching and fledging for each nest-box, such that parents would be maximally motivated to return to their box. For each trial, the experimenter set up a camouflaged hunting hide within 10–20 m of the nest-box. The hunting hide was placed in the same location for every trial at each nest-box. A camcorder (Panasonic HC-V130) wrapped in camouflage tape was placed on the ground adjacent to the hide to record the trial. The experimenter then swapped the habituation apparatus for the motorized apparatus and a test stimulus. The experimenter watched the trial from inside the hide and triggered the remote control when a jackdaw approached within 2 m of the nest-box, noting the precise time (in seconds) when jackdaws were in view of the nest-box, approached the nest-box, made defensive scolding calls, entered or exited the nest-box.

An approach was defined as a jackdaw coming within 2 m of the nest-box, from an angle where the stimulus would be visible. The perching locations that fell within the 2 m radius were determined ahead of the trial for each nest-box and kept constant across trials at that box. Once a jackdaw approached, the motor was triggered for 10 s, either creating movement or not, depending on the condition. The jackdaw receiving the initial trigger was treated as the focal individual. The trial lasted for 20 min after the initial approach; a time limit substantially longer than the mean time of birds in these populations to enter their nest-boxes after approaching a novel object at their nest [28]. The motor was re-triggered every time a new jackdaw came into view and approached. There were no differences between trial types in the number of times the motor was triggered (electronic supplementary material, table S1). If the focal individual remained in sight of the experimenter, then the motor was not re-triggered when it re-approached because it was known to have experienced the motor movement before. If no jackdaws approached within the maximum time of 80 min, the trial was discarded. Discarded conditions were repeated as a new pair of *Moving* and *Non-moving* trials at the end of the run of trials, using the other stimulus from that category. If no jackdaws approached within the maximum time on two consecutive trials, no further trials were conducted at that nest-box and the failed trials were removed from the analysis. There were 14 trials in which a non-focal individual entered the nest-box before the focal individual approached. These trials were only kept in the analyses if the non-focal individual exited the nest-box before the focal individual was in view of the nest-box from the experimenter's perspective. Doing so was warranted because when these 14 trials were excluded and the data re-analysed, similar results were found (electronic supplementary material).

All trials were subsequently video coded to verify the in-person observations, a subset of which (15%) were additionally coded by an observer blind to the results. Looking time was not used as a response variable because it cannot be assessed accurately with species such as birds that have a large visual field without training them to use a peep-hole (e.g. [14]); such training was not possible prior to testing wild birds. Instead, it was noted if birds startled when the motor was triggered by responding fearfully, as indicated by hopping backwards (i.e. a corvid-typical fear behaviour [29]), flying away, or scolding the object. Hops where their wings did not open (i.e. a general repositioning instead of a fear display) were not included. Birds' nest-box entrance time was also noted, and if they did not enter within 20 min of the trigger, they were assigned a maximum entrance time of 1200 s. Prior research with jackdaws indicates that latency to approach and enter a nest-box can be used as a threat response measure [24,30]. The total number of scolding calls (a defensive alarm call used to recruit others and drive away potential threats [31,32]) made from the time of the trigger to the end of the trial was recorded to determine if jackdaws attempted to defend their nest when the stimulus was presented. Inter-coder reliability was assessed with a one way intraclass correlation coefficient [33] and was extremely high (ICC (1) = 0.96, CI = 0.91–0.98, *p* < 0.001).

### 2.3. Analysis

All statistics were conducted in R [34]. The lme4 [35] package was used for mixed models, and the survival package [36] was used for the Cox proportional hazards regression. The influence of stimulus type and movement was assessed for each response variable (startle response to trigger, post-trigger entrance latency and defensive scolding). Study site, trial pair number, within-pair order and whether a non-focal jackdaw had entered before the motor was triggered were all included as additional explanatory variables.

#### 2.3.1. Startle response to trigger

The factors that influenced jackdaws’ fearful responses towards the trigger (*N* = 27 pairs of birds, 143 trials) were assessed with a generalized linear mixed model (GLMM) with a binomial error structure indicating whether or not (1,0) a jackdaw was startled at the trigger (i.e. flew away, fear hopped or scolded), including nest-box as a random factor. Terms were removed from the model if their exclusion failed to increase Akaike information criterion (AIC) values by at least 2.0. See electronic supplementary material, table S2, for the model selection process.

#### 2.3.2. Post-trigger entrance latency

The birds’ latency to enter the nest-box after the first trigger served as a measure of their hesitancy to turn their back on the different stimulus types (*N* = 27 pairs of birds, 143 trials). Entrance latency was analysed with a Cox proportional hazards survival analysis because latencies were truncated by the end of the trial time. Observations were clustered by nest-box to account for the fact that the responses within individual nests would be correlated. Interactions arising from the final model were investigated by conducing *post hoc* survival models on subsets of the data, using only significant terms from the main model. Bonferroni corrections were applied to *p*-values from *post hoc* survival tests to account for multiple testing.

#### 2.3.3. Post-trigger defensive scolding

The distribution of the number of scolds produced per trial was highly right skewed, with many individuals never scolding, some individuals only scolding once and other scolding as many at 88 times in a single trial. To deal with the skewed data, whether or not jackdaws scolded after the trigger was transformed to a binomial variable (scolds 1/0). Scolding behaviour was assessed with a GLMM with a binomial error structure, with nest-box as a random factor. Terms were removed from the model if their exclusion failed to increase AIC values by at least 2.0. See electronic supplementary material, table S4, for the model selection process.

## 3. Results

We tested wild jackdaws across a total of 27 nest-boxes—15 of which received a *Moving* and *Non-moving* presentation of all three stimuli on 6 separate days and 12 of which had at least one *Moving* and *Non-moving* stimulus pair—resulting in a total of 143 trials. In 14 of those 143 trials, a non-focal bird entered the nest-box ahead of the focal bird (i.e. the bird that experienced the initial trigger), which was accounted for in later analysis as a term in statistical models (also see electronic supplementary material, for additional analyses without these 14 trials). A summary of the sample sizes and descriptive statistics for the three stages of analysis can be found in table 1.

**Table 1.** Summary table of response measures.

response to trigger^a^	*N*	percentage of birds that startled
condition		
moving stimuli	71	57.7%
non-moving stimuli	72	22.2%
post-trigger defensive scolding	*N*	percentage of birds that scolded
condition		
moving	71	26.7%
non-moving	72	13.9%
bird	47	25.5%
snake	47	25.5%
stick	49	10.2%
post-trigger latency to enter nest-box	*N*	mean (±s.e.) time delay to enter the nest in seconds
condition		
bird		
moving	22	659 (±120)
non-moving	25	440 (±99)
* *snake		
moving	24	918 (±87)
non-moving	23	554 (±95)
stick		
moving	25	794 (±90)
non-moving	24	274 (±80)

^a^The response to trigger measure did not differ between stimulus types of the moving and non-moving conditions.

### 3.1. Startle response to trigger

**Figure 2.**
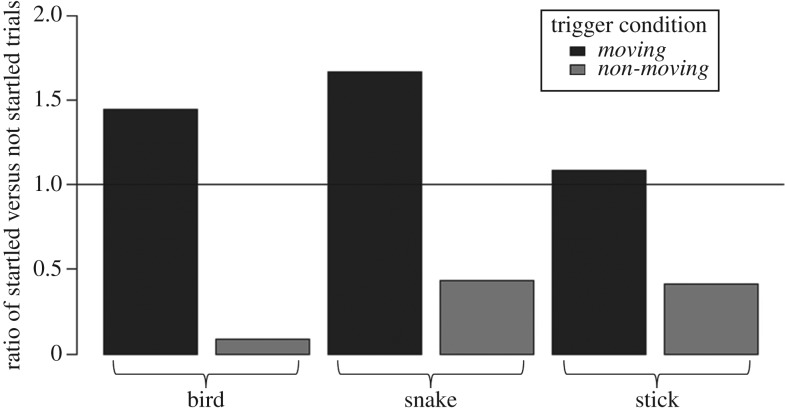
Startle response to motor trigger. Ratio of the number of trials where a jackdaw was startled by the motor to the number of trials where the jackdaw was not, broken down by stimulus type and movement condition. A ratio of one means that an equal number of birds were and were not startled when presented with that stimulus (as represented by the horizontal line), and a low value means that very few birds were startled compared to the number that was not startled. Jackdaws were more likely to react towards the trigger in *Moving* conditions than *Non-moving* conditions, regardless of stimulus type.

Jackdaws were more likely to startle (jump, produce scolding calls, or fly away) in response to the trigger of the remote-controlled motor in conditions where the stimulus moved than when it did not move (Binomial GLMM, *n* = 143, Est. = 1.98 ± 0.47, ΔAIC = −20.6). This response to movement occurred independently of the stimulus type, as there was no interaction between stimulus and movement condition (figure 2; electronic supplementary material, table S2). In all trials where a bird startled (*n* = 57), they always returned to inspect the stimulus before the end of the trial.

### 3.2. Post-trigger entrance latency

**Figure 3.**
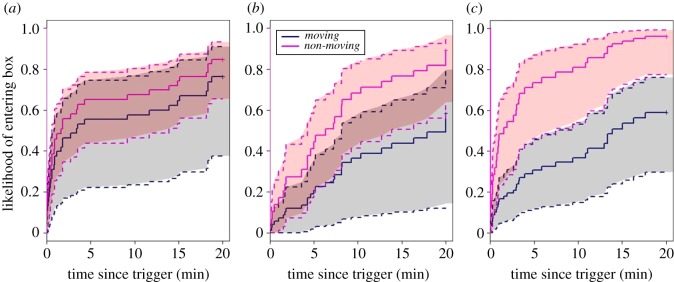
Post-trigger latency to enter nest-box. Inverted survival curves showing the likelihood that jackdaws entered the nest-box over time after the motor was triggered. Jackdaws were presented with either a *Moving* or *Non-moving*: (*a*) bird; (*b*) snake; (*c*) stick. Jackdaws were equally likely to enter the box over time when the bird was moving or stationary, they were mildly deterred from entering by the moving snake and the largest difference occurred between the moving and non-moving stick. Dotted lines denote confidence intervals, which overlap in the bird and snake conditions (*a*,*b*) but do not overlap in the stick condition (*c*).

There was an interaction between the effect of movement and the type of stimulus, indicating that the effect of movement differed between stimuli (Table 2). *Post hoc* tests (with Bonferroni corrections) investigating these relationships revealed that jackdaws were equally quick to enter their nest-box after being presented with a *Moving* or *Non-moving* bird (Cox proportional hazards model, *n* = 47 observations, 43 events, *B* ± s.e. = −0.265 ± 0.40, *z* = −0.265, *p* = 1.00). By contrast, they delayed their entrance after seeing the *Moving* snake and stick in comparison to their *Non-moving* conditions. Notably, movement had a substantially larger effect for stick trials than for snake trials (stick, *n* = 49 observations, 36 events, *B* ± s.e. = −1.29 ± 0.31, *z* = −4.05, *p* < 0.001; snake, *n* = 47 observations, 29 events, *B* ± s.e. = −0.924 ± 0.46, *z* = −2.59, *p* = 0.029; figure 3). The cause of this difference stemmed from the *Non-moving* conditions; jackdaws were more likely to enter their nest-boxes when presented with a stick than a snake (*n* = 47 observations, 40 events, *B* ± s.e. = 0.676 ± 0.32, *z* = 2.46, *p* = 0.037). Responses were indistinguishable between exemplars of each stimulus type (electronic supplementary material).

**Table 2.** Cox proportional hazards model, *n* = 143 observations, 99 events for post-trigger latency to enter the nest-box. Variable level listed within parentheses. Statistically significant effects are in italics. *B* is the coefficient or hazard ratio. A larger value for *B* indicates an increased probability that they will enter the box. For example, at each time point (in seconds) jackdaws which had startled at the trigger were 80% less likely to enter than jackdaws which had not startled. Bird stimuli serve as the reference category for stimulus type.

	*B*	±s.e.	*z*	*p*
movement (*non-moving*)	−0.30	0.37	−0.92	0.356
stimulus				
snake	−0.03	0.33	−0.10	0.922
* *stick	*0.72*	*0.32*	*2.72*	*0.006*
trial order (*moving* first)	0.18	0.21	0.94	0.346
trial set	−0.14	0.12	−1.44	0.149
area (Cornwall)	0.45	0.22	1.36	0.175
non-focal entered (Y)	*0.66*	*0.32*	*2.73*	*0.006*
startled at trigger (Y)	*−0.8*	*0.25*	*−2.39*	*0.017*
stimulus * movement interaction				
*moving* * snake	−0.51	0.53	−1.49	0.136
* moving* * stick	*−1.03*	*0.51*	*−3.05*	*0.002*

Asterisk (*) denotes an interaction.

This analysis also revealed that jackdaws that startled at the trigger took longer to enter regardless of condition (*z* = −2.39, *p* = 0.017; table 2), suggesting that being startled elicited a fear response in those subjects. Finally, the focal jackdaw was more likely to enter if a non-focal individual entered previously (*z* = 2.73, *p* = 0.006). However, when these 14 trials were excluded, the same interaction patterns between movement and stimulus type were observed (electronic supplementary material).

### 3.3. Post-trigger defensive scolding

**Figure 4.**
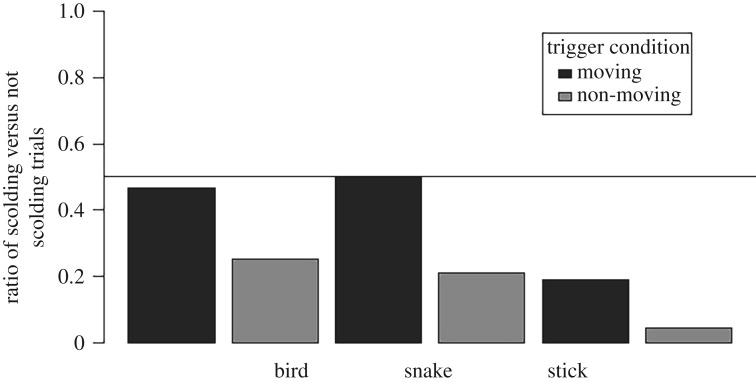
Post-trigger defensive scolding. Ratio of the number of trials where a jackdaw scolded the stimulus after the trigger to the number of trials where the jackdaw did not, broken down by stimulus type and movement condition. A ratio of one means that an equal number of birds did and did not scold when presented with that stimulus. The line pictured at 0.5 indicates conditions where half as many jackdaws scolded than those that did not. Despite many jackdaws not exhibiting scolding behaviour, significant patterns still emerged. Jackdaws were more likely to scold stimuli in *Moving* conditions than *Non-moving* conditions and were equally likely to scold the bird and the snake conditions, while they were significantly less likely to scold when presented with either type of stick.

Many jackdaws did not scold in any of the experimental conditions (*N* = 14 of 27 nests tested), but overall scolding was more likely after being presented with a moving compared to a non-moving stimulus (Binomial GLMM, *n* = 143 trials, Est. = 1.40 ± 0.69, ΔAIC = −3.7). Jackdaws were equally likely to scold bird and snake stimuli (Binomial GLMM, *n* = 143, Est. = −0.17 ± 0.61, ΔAIC = −3.8) and less likely to scold sticks than either the snake or bird (Binomial GLMM, *n* = 143, Est. = −1.79 ± 0.76, ΔAIC = −3.8). There was no evidence for an interaction between stimulus type and movement on scolding behaviour (figure 4; electronic supplementary material, table S4).

## 4. Discussion

The primary evolutionary function of animacy categorizations is widely assumed to relate to intra-specific social behaviour [2,5,37]. We investigated an alternative possibility; that judgements of object animacy may play a role in assessing potential threats in the environment. We found that wild jackdaws were equally startled by the sudden movement of stimuli representing animate or inanimate objects and were most defensive towards animate objects that should be expected to respond to alarm calls (e.g. snakes and birds). However, when presented with an independently moving stick that violates expectations of animacy, jackdaws were not overly defensive, but were more likely to hesitate and delay entering their nest. These results suggest that wild jackdaws may judge the animacy of objects in their environment when assessing unknown or potential threats, that they respond defensively towards expected animate objects and show caution towards unexpected animate movement.

Jackdaws were startled to a similarly high degree by the sudden movement of all stimulus types at their nest-boxes. Meanwhile, the *Non-moving* conditions only startled a small percentage of birds. A startle response would not be expected as birds approached in *Non-moving* trials because there was no change in any of the stimulus types at the exact time of the trigger, apart from the sound of the motor. Since movement is a common cue animals use to identify predators [7,13], it is therefore not surprising that all *Moving* stimuli evoked a rapid startle response. However, the type of stimulus presented influenced jackdaws' level of defensive behaviour (scolding alarm calls) at their nest. Jackdaws were more likely to alarm call in the presence of naturally animate stimuli, i.e. a snake or a bird, than in the presence of a stick at their nest-box. Although a small passerine bird should not be threatening, usually these birds would leave a nest-box area upon the approach of a larger, dominant corvid species. The fact that the small bird did not leave at the jackdaws’ approach, which would be highly unlikely if it were alive, may explain why the bird stimulus elicited defensive scolding behaviours at a similar level to when a simulated predator was present.

Across all stimulus types, jackdaws that were startled by movement took longer to enter their nests, but even in cases where birds fled at the movement of the trigger, they always came back to the nest-box and directed attention towards the stimulus before the end of the trial. This suggests that the delays in nest-box entrances may serve an information gathering function, as violations of expectations are expected to do [38], perhaps in this case to determine the nature of the stimulus and source of its movement. In the *Moving* versus *Non-moving* bird conditions, there was no difference in the propensity of jackdaws to delay entering their nest-boxes, suggesting that movement in the bird, even if slightly unnatural in its presentation, did not provide any additional information that warranted inspection. In comparison to its *Non-moving* condition, the moving snake may have indicated a heightened threat because birds delayed entering their nest-boxes and demonstrated defensive scolding behaviours. Meanwhile, the effect of movement on entrance times was greatest for the stick conditions, suggesting that there was a particular need to gather information when presented with a suddenly moving stick. Coupling this delay with the fact that jackdaws did not actively defend their nest during stick conditions suggests that sensitivity to apparent violations of animacy may serve to promote further information gathering about *unknown* risk. In this case, the moving stick was not deemed a known threat to be met with scolds, but instead movement appeared to be unexpected.

Regardless of whether jackdaws' extended delay in moving stick conditions stemmed from the novelty of seeing a self-propelled stick, something about the moving stick prompted comparatively greater caution at the nest-box than other types of movement. The results suggest that the differences between stimuli types are likely to be due to some level of object categorization because jackdaws’ responses were indistinguishable between the two different exemplars of each stimulus type (stick, snake or bird). However, although our results showed discrimination between object types on the basis of animacy, our results do not shed light on how such categorization would arise; a process which could vary by species. In theory, jackdaws’ animacy categories could reflect ‘core knowledge’—that is, evolved cognitive representations of object properties that are present from birth [11]. Such representations are often suggested to underlie animacy categorizations in early life for humans [11,39] and other species [40]. In birds especially, these early representations are proposed to aid in filial imprinting [9]. While animacy concepts have not been tested in altricial (non-imprinting) species including members of the corvid family, there is evidence that, at least in the case of the Eurasian jay (*Garrulus glandarius*), young corvids show expectations about other properties of objects, such as rules governing support, at six months of age, and that this core knowledge emerges even in the absence of experience with the testing stimuli [14]. Therefore, animacy categories could develop alongside other types of physical cognition in altricial species as well as precocial ones.

An alternative, and arguably simpler, potential explanation for our findings is that jackdaws’ object categorizations could be formed by associative learning from experience with animate and inanimate stimuli that then allows for generalizations to other stimuli with similar characteristics. If this is the case, then jackdaws’ hesitancy around moving sticks could be rooted in a reaction to the novelty of the type of movement exhibited by sticks in our experiment. While sticks may naturally sway in the wind, they do not suddenly make directional movements in a horizontal plane as our moving stick did. The prolonged hesitancy of jackdaws in response to the motorized, moving stick could thus reflect the violation of an associatively learned rule: this type of object does not normally move of its own accord, movement as we presented it was novel, and novelty should prompt greater caution. Via this route of associative learning, expectations about each type of inanimate object in the environment could arise independently or be generalized across object types based on some perceptual feature. The ‘core knowledge’ and ‘associatively learned categories’ hypotheses are not mutually exclusive, because learning could, in principle, build on core knowledge concepts [41]. The extent to which experience is needed for the expression of animacy distinctions in wild animals could be tested in future via presentations of familiar versus novel types of stimuli with which individuals lack experience.

Regardless of how animacy categories are formed, the potential existence of these categories in wild corvids suggests they may play an important role in threat-based decision-making in the wild. One might speculate that this phenomenon should be taxonomically widespread, as many animals respond selectively to motion in the context of predatory threats [7,13]. Therefore, Aristotle's view of a world divided into animate and inanimate objects is one which we may share with a wide range of species.

## Supplementary Material

Additional analyses, tables and figures

## Supplementary Material

Video of triggered bird movement

## Supplementary Material

Data for Greggor et al., RSOS

## Supplementary Material

Code for analysis in Greggor et al., RSOS

## Data accessibility

All data and R code used for the analysis of this publication can be found in the electronic supplementary material.
