# Supplementary material for: Wild jackdaws are wary of objects that violate expectations of animacy
Source: R Soc Open Sci. 2018 Oct 24;5(10):181070. doi: 10.1098/rsos.181070 (PMC6227974; doi:10.1098/rsos.181070)
Supplement: Additional analyses, tables and figures [file rsos181070supp1.pdf]

Electronic Supplemental Material for Greggor, McIvor, Clayton, Thornton, "Wild jackdaws are wary of objects that violate expectations of animacy"

Additional analyses

Since jackdaws' nestbox entrance latencies differed by stimulus type, differences within stimuli types were compared to ensure that responses to both of the exemplars were similar enough to deem them a category. No differences in latency to enter the nest box were found between exemplars of any stimulus type (Student's t-test, birds: $n = 47$, $t = -1.21$, $df = 39.51$, $p = 0.234$; snakes: $n = 47$, $t = 0.249$, $df = 42.04$, p-value = 0.805; sticks: $n = 49$, $t = 0.289$, $df = 38.19$, p-value = 0.774).

In 14 trials, a non-focal individual entered the nestbox before the focal individual, but exited prior to the arrival of the focal individual. An analysis was conducted without these 14 trials to ensure that the results were not overly swayed by these data points. When these 14 trials were excluded, the same interaction patterns between movement and stimulus type were observed (Figure S1, Table S2).

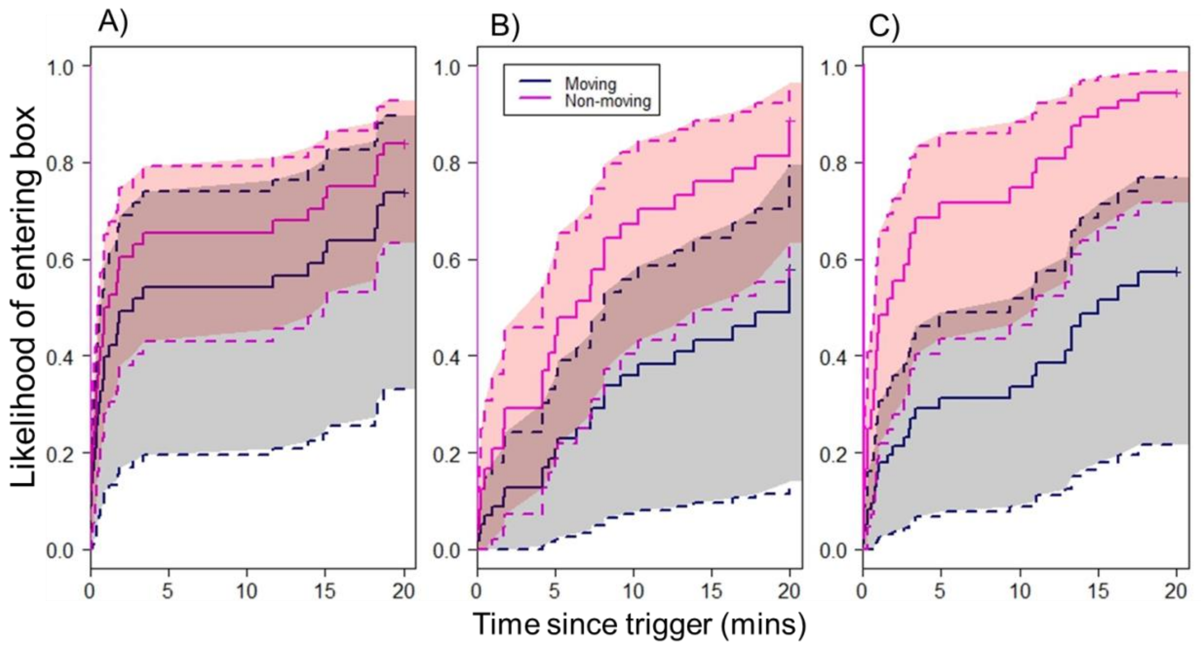

Fig. S1 Post trigger avoidance with restricted sample size Likelihood that jackdaws entered the nest-box over time after the motor was triggered, discarding all trials in which a non-focal individual entered the nest-box before the focal jackdaw. The panels represent the *Moving* and *Non-moving*: A) bird stimulus; B) snake stimulus; C) stick stimulus. The same trends are apparent as the full sample size (Figure 3, main text)

Trial type	Mean No. times motor triggered (±SE)
Moving	2.211 (±0.15)
Non-Moving	1.917 (±0.12)
Bird	2.085 (±0.17)
Snake	2.106 (±0.19)
Stick	2.000 (±0.13)

Table S1. Summary of trigger events per trial type. The same motor was triggered, regardless of condition, and it was triggered each time a bird approached the nest box. No differences arose between the trial types in the number of times the motor was triggered.

Model	ΔAIC
Startle(Y/N) ~ Movement +Stimulus + Field site + Trial set + Trial order + Movement:Stimulus + Non-focal entered + (1|Box)	0.0
Startle(Y/N) ~ Movement +Stimulus + Field site + Trial set + Trial order + Movement:Stimulus + (1|Box)	-1.4
Startle(Y/N) ~ Movement +Stimulus + Field site + Trial set + Trial order + (1|Box)	-0.1
Startle(Y/N) ~ Movement +Stimulus + Field site + Trial set + (1|Box)	-0.9
Startle(Y/N) ~ Movement +Stimulus + Field site + (1|Box)	+1.2
Startle(Y/N) ~ Movement +Stimulus + (1|Box)	+1.1
**Startle(Y/N) ~ Movement + (1|Box)**	**+1.0**
Startle(Y/N) ~ (1|Box)	+20.6

Table S2. Model selection process of binomial GLMM on jackdaws' startle response. Removing the Movement variable increased the model AIC by more than 20. Final model is highlighted in bold.

	B	±SE	*z*	p
Movement (*Non-moving*)	-0.5	0.38	-1.32	0.188
Stimulus				
Snake	0.72	0.46	2.32	0.021
Stick	0.34	0.42	1.22	0.223
Trial order (*Moving* first)	-0.32	0.23	-1.5	0.133
Trial set	0.13	0.13	1.32	0.188
Startled at trigger (Y)	0.68	0.26	1.83	0.068
Area (Cornwall)	-0.50	0.23	-1.45	0.147
Stimulus * Movement interaction				
Moving * snake	-0.53	0.56	-1.46	0.144
***Moving* * stick**	-0.84	0.54	-2.22	0.027

Table S3. Cox proportional hazards model, n = 129 observations, 86 events for post-trigger latency to enter the nest box (restricted dataset excluding trials where non-focal bird entered first). Variable level listed within parenthesis. Statistically significant effects are in bold. B is the coefficient, or hazard ratio. The bird is the reference category for stimulus type. * denotes an interaction.

Model	ΔAIC
Scolds(Y/N) ~ Movement +Stimulus + Trial set + Non-focal entered + Field site + Movement:Stimulus + Trial order + (1|Box)	0.0
Scolds(Y/N) ~ Movement +Stimulus + Field site + Trial set + Non-focal entered + Field site + Movement:Stimulus + (1|Box)	-2.0
Scolds(Y/N) ~ Movement +Stimulus + Trial set + Non-focal entered + Field site + (1|Box)	-3.5
Scolds(Y/N) ~ Movement +Stimulus + Trial set + Non-focal entered + (1|Box)	-1.2
Scolds(Y/N) ~ Movement +Stimulus + Trial set + (1|Box)	-1.2
**Scolds(Y/N) ~ Movement +Stimulus + (1|Box)**	**+0.1**
Scolds(Y/N) ~ Movement + (1|Box)	+3.8
Scolds(Y/N) ~ (1|Box)	+3.7

Table S4. Model selection process of binomial GLMM on jackdaws' defensive scolding response. Removing the Stimulus and Movement variables both increased the model AIC by more than 2. Final model is highlighted in bold.
